# Social and health care utilization before and after opioid initiation in home care recipients with and without dementia: a nationwide register-based cohort study

**DOI:** 10.1186/s12877-025-06550-z

**Published:** 2025-11-03

**Authors:** Heidi Mörttinen-Vallius, Jaana Keto, Esa Jämsen, Miika Linna

**Affiliations:** 1 Faculty of Medicine and Health Technology, University of Tampere, Tampere, Finland; 2 Faculty of Medicine (Department of Oral and Maxillofacial Disease), University of Helsinki, Helsinki, Finland; 3 Faculty of Medicine (Clinicum), University of Helsinki, Helsinki, Finland; 4 Department of Geriatrics, Helsinki University Hospital, Helsinki, Finland; 5 Aalto University, Espoo, Finland; 6 University of Eastern Finland, Kuopio, Finland

**Keywords:** Older people, Dementia, Health care service utilization, Home care, Opioids

## Abstract

**Background:**

Opioid use is associated with increased health care service utilization but home care recipients and patients with dementia have been mostly ignored in earlier studies, although changes in their health status can have dramatic cost consequences. This study examined social and health care utilization and costs among older home care recipients before and after opioid initiation, with persons with dementia as a subgroup of interest.

**Methods:**

This retrospective nationwide register-linkage study included Finnish regular home care recipients aged ≥ 65 years with opioid initiation between 1st March 2015 and 31st December 2016. Recipients with health care contacts due to cancer and palliative care were excluded.

Incidence rate ratios of social and health care service use during the observation period starting one year before and ending one year after opioid initiation were calculated using nationwide register data. The utilization costs of various social and health care service categories were compared before and after the opioid initiation. Recipients with and without dementia were analysed separately.

**Results:**

Home care recipients had 1.76 (95% CI 1.75 − 1.78) times more inpatient days, 1.31 (1.26 − 1.36) times more emergency care admissions, and recipients without dementia had more outpatient service use (secondary care 1.10 [1.06 − 1.13], primary care 1.06 [1.04 − 1.07]) after opioid initiation compared to the preceding year. Home care service use decreased (0.85 [0.85 − 0.85]) in recipients with dementia but increased in those without (1.08 [1.08 − 1.08]). Of the recipients, 20.8% were admitted to long-term residential care during the follow-up year. The mean annual total costs per recipient were 21% higher during the year following opioid initiation compared to the preceding year. A peak in the costs, consisting largely of inpatient costs, was observed about a month before opioid initiation, after which costs showed a declining trend but remained above the baseline level.

**Conclusions:**

Recipients’ monthly health care resource use started to increase already before opioid initiation, after which monthly expenditures declined steadily, which may reflect mostly recipients’ worsened health status before opioid initiation. Most of the expenditures arose from housing services. The relationship between opioid use and utilization of residential care and home care services should be further examined.

**Trial registration:**

Not applicable (a retrospective register-based study).

**Supplementary Information:**

The online version contains supplementary material available at 10.1186/s12877-025-06550-z.

## Background

About one-fourth of older adults in the US [1] and nearly one-fifth in the Nordic countries [2] are prescribed opioids each year. Community-dwelling, mostly middle-aged opioid users have more hospitalizations, emergency department visits, and outpatient contacts, than users of non-steroidal anti-inflammatory drug (NSAID) [3]. Health care service utilization seems to increase with increasing opioid dose and duration of use [4–6]. Moreover, higher health care expenditures have been reported even in opioid users without or with only mild pain interference compared to non-users with moderate to severe pain interference [7]. In home care setting, long-term opioid users have been reported to have a higher number of outpatient contacts but not inpatient days compared to non-users [8]. However, frail older adults have been mostly ignored in earlier studies on health care service utilization among opioid users. Furthermore, a major component of the social and health care costs of older adults – housing services – is typically not included in the analyses.

Home care, i.e., regular home visits by a nurse, is a way to extend the time an older adult can live at home before moving on to residential care. The transition to more heavily supported housing services is typically triggered by a change in the health status of a home care recipient. Painful conditions are such change, but information about pain and physical function are typically not available in health registers. Opioid initiation instead can be identified from administrative health care registers and can be used as proxy for significant change in health status. While opioids may have a positive short-term effect on a home care recipient’s health when used to treat acute pain, they can also lead to social and health care service use due to potential adverse events.

People with dementia are a frail subgroup of older adults whose social and health care use profile differs from that of other older adults. Social and health care expenditures are higher in older adults with dementia compared to matched persons without cognitive impairment both before and after a diagnosis [9–11]. The association between dementia and opioid use has varied in previous studies, but in home care setting dementia has been related to a lower rate of opioid use [12–14]. Previous studies in geriatric settings have indicated that the patterns of opioid use and factors associated with persistent use (such as chronic diseases, history of fractures, disabilities in activities of daily living, and use of psychotropics) may differ between opioid users with and without dementia, which may affect their social and health care service use [14–16].

The objective of this study was to examine social and health care utilization and costs, including transitions to long-term residential care, in a nationwide unselected sample of older home care recipients before and after opioid initiation for non-cancer pain, with dementia patients as a subgroup of interest.

## Methods

### Study population

**Fig. 1:**
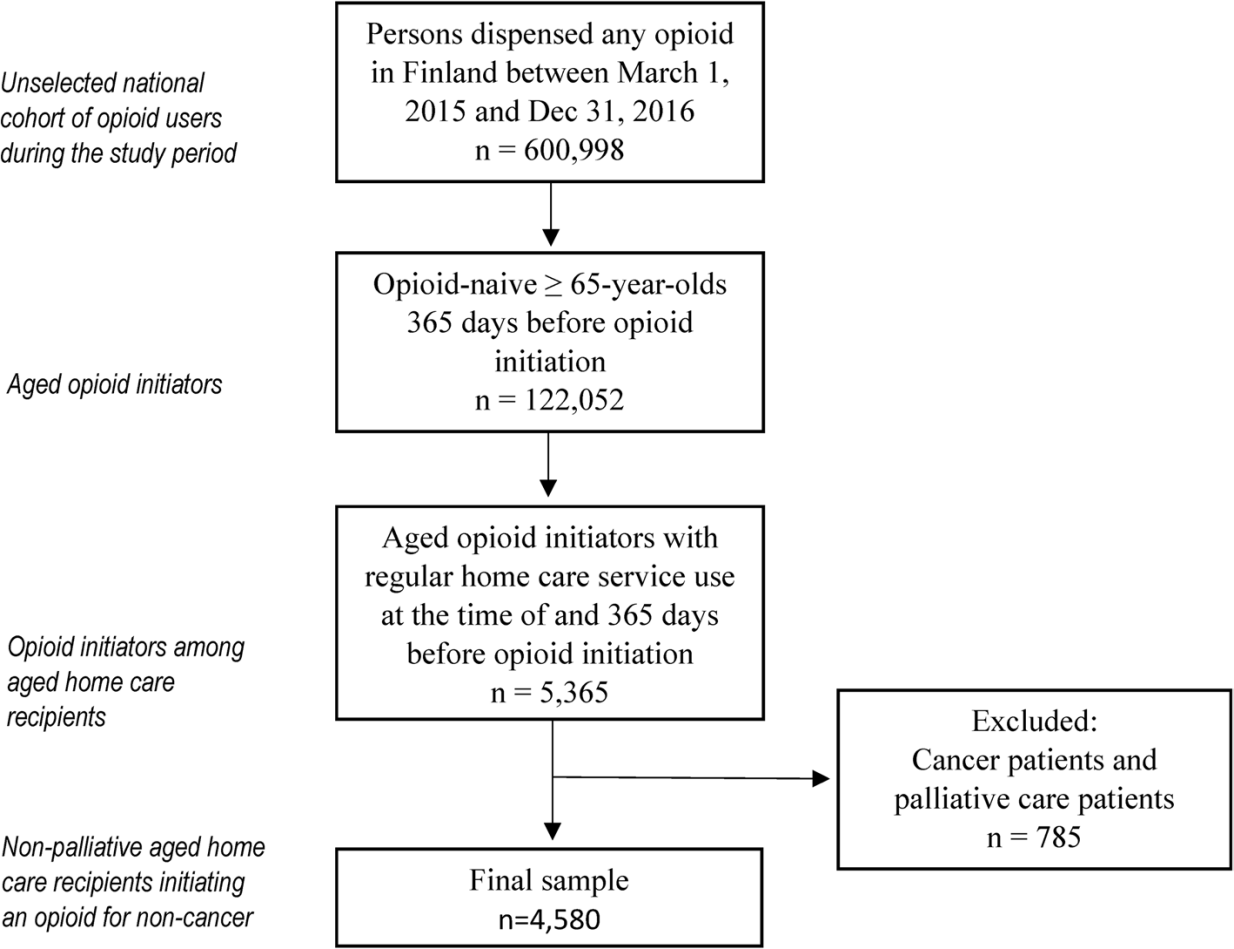
Sample selection

The present study was based on a nationwide register-linkage based cohort of all inhabitants in Finland who had at least one opioid purchase between 1 st January 2009 and 31 st December 2017 [17]. A sub-cohort of regular home care recipients who were dispensed any opioid between 1 st March 2015 and 31 st December 2016 was extracted from the original cohort of all Finnish opioid users (Fig. 1). The date of a first opioid purchase during the study period was defined as each person’s index date. Data on regular home care use were available since 1 st January 2014, which set the opioid users’ enrolment window. According to the Finnish Institute of Health and Welfare’s definition used in the national Health Care Register, persons were regarded as regular home care recipients if they had at least six nurse visits within the preceding 60 days. This definition was used as an inclusion criterion both at the index date and at 365 days prior to the index date. Persons with less visits were excluded.

**Fig. 2:**
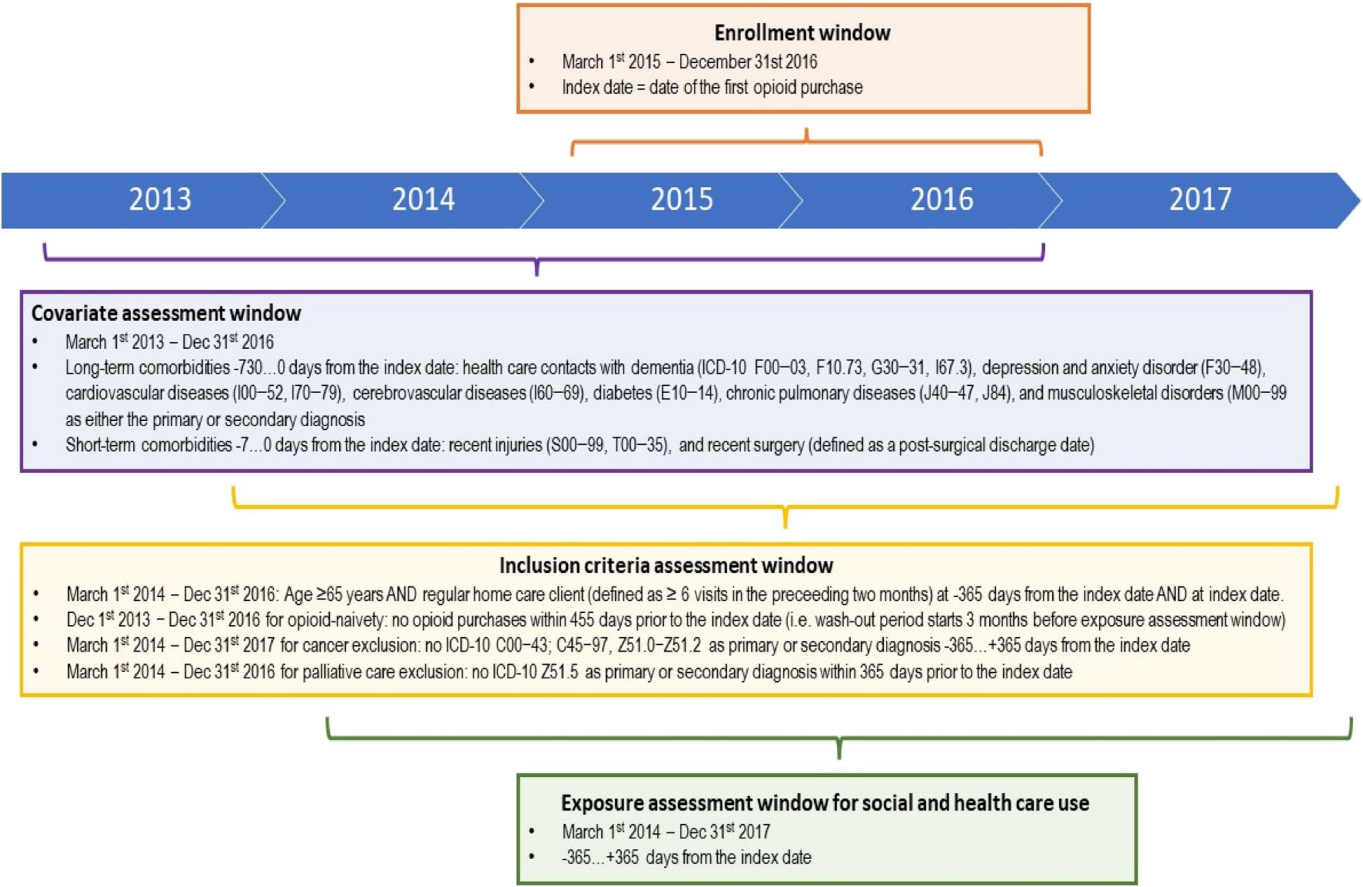
Study design

The observation period of all recipients started from one year before and ended one year after the index date (Fig. 2). All persons had to be ≥ 65 years old at the beginning of the observation period. To ensure that recipients were not using opioids during the one-year period prior to the index date, they could not have any opioid purchases within 455 days before the index date. This wash-out period also minimised the number of patients with chronic pain-related conditions. The period of 455 days (= 365 + 90) was based on the fact that in Finland prescription drugs, including all opioids, can be dispensed for a maximum of three months use at a time.

To form a cohort of home care recipients using opioids for non-cancer pain and non-terminal conditions, recipients with a health care contact due to cancer, chemotherapy, or radiotherapy (the International Classification of Diseases 10th revision [ICD-10] codes C00 − 97, except basal cell carcinoma, and Z51.0 − Z51.2) during the observation period were excluded. Also, recipients with the ICD-10 code Z51.5 (palliative care) during the one-year period before the index date were excluded.

### Data sources

Data were gathered retrospectively from nationwide Finnish social and health care registers and linked using the personal identification number of each inhabitant. Data obtained from different registers and the state agencies responsible of these registers are presented in Table 1.

The data owners granted permissions to use the data for research purposes and pseudonymised it before passing the data to the investigators. According to the current national and European Union legislation, retrospective register-based secondary data can be used for research without the participants’ informed consent.

**Table 1:** Data sources used in register linkage and information obtained for the present study

Data source	Register holder	Information obtained
National prescription register	The Social Insurance Institution of Finland	Dispense date and product code of the dispensed opioid, which carries information on opioid type (ATC), dose strength, formulation, and package size
Primary and secondary health care register	Finnish National Institute for Health and Welfare	Dates and diagnoses (ICD-10 codes) and interventions recorded for each outpatient visit and inpatient discharge event in both primary and secondary care. Home care nurse visits.
Social care register	Finnish National Institute for Health and Welfare	Dates in long-term residential care
Population register	Finnish Population Register Centre	Date of birth and place of residence
Cause of death register	Statistics Finland	Dates and causes of deaths
Register of completed education and degrees	Statistics Finland	Education level

### Variables and outcomes

#### Characteristics of home care recipients

The following characteristics for the cohort at the index date are reported: gender, age (mean, percentage of > 75-year-olds), education level (primary, secondary, or university degree), persons residing in cities of > 80,000 inhabitants, number of home care nurse visits per day during the year preceding the index date, and the presence of common chronic diseases that may affect opioid use and social and health care use. The reported chronic diseases were dementia (ICD-10 F00 − 03, F10.73, G30 − 31, or I67.3), depression and anxiety disorders (F30 − 48), cardiovascular (I00 − 52, I70 − 79) and cerebrovascular diseases (I60 − 69), diabetes (E10 − 14), chronic pulmonary diseases (J40 − 47, J84), and musculoskeletal disorders (M00 − 99), appearing as a primary or secondary diagnosis within two years before the index date. Recent surgery was defined as a post-surgical discharge date appearing within one week before the index date. Recent injuries (S00 − 99, T00 − 35) were defined similarly.

#### Opioid use

The opioid analgesics (N02A according to the WHO Anatomical Therapeutic Chemical classification) available in Finland during the study period were morphine, hydromorphone, oxycodone, fentanyl, buprenorphine, codeine (in combinations with acetaminophen or NSAIDs), and tramadol. The first five were defined as strong opioids. The national prescription register includes all dispensed opioids for out-patient use. To account for potency differences between different types of opioids, we express opioid usage as oral morphine milligram equivalents (OMEQ), based on standard equianalgesic ratios [18]. The oral morphine milligram equivalents (OMEQ) used by each person cumulatively during the inspection year were calculated based on opioid type, dose, formulation, and package size of each opioid purchase made during the observation year. The initial amount of opioids (OMEQ) dispensed from the pharmacy at the index date, and the cumulative consumption during the one-year period after opioid initiation are reported as a mean for the inspected population. The pattern of opioid use was categorized either as short-term (opioids dispensed only within the first three months) or extended (opioids dispensed also within 3 − 12 months of the index date).

#### Social and health care use

Social and health care service use and cumulative costs are presented over an observation period of one year both prior to and after the index date. The percentage of recipients who died or were permanently admitted into long-term residential care (defined as an admission lasting ≥ 3 months) within one year of the index date are reported. Health care service use covers both primary and secondary care and includes inpatient days, admissions to emergency care, primary and secondary care outpatient visits either in a health care facility or at home care recipient’s own home, and primary care phone and virtual contacts. In primary care, all contacts by different health care professionals (physicians, registered nurses, physiotherapists etc.) are included. In secondary care only data on outpatient contacts by physicians were available.

The reported social service use consists of home care nurse visits and days in long-term residential care, i.e., round-the-clock care in nursing homes and assisted-living facilities for the aged. Home care nurse visits include, based on a recipient’s personal needs, assistance with activities of daily living, medical care at home, and administering of medication. Some home care recipients may have had an informal caregiver (usually a spouse or a child) who is entitled statutory leave days. In these recipients, the median number of long-term residential care days (but not long-term residential care admissions) includes these caregivers’ statutory leave days, during which the recipient has been in short-term care in a residential care facility. Public social and health care services are tax-financed in Finland and were run by municipalities at the time of the observation period. Nurse visits are the main service type of public home care services in Finland, and if a person does not need regular nurse visits but only support services such as meal or cleaning services, they are often purchased from private sector or are managed by family members. Private health care use among home care recipients in Finland is rare and is not included in the reported outcomes.

The reported costs are actual production costs of delivering care, including all fixed and changing costs. The costs for secondary health care use are based on the Diagnosis Related Group of the Nordic countries (NordDRG) where all outpatient visits and inpatient care periods of each patient are assigned to economically homogenous groups based on ICD-10 codes, codes for diagnostic and treatment procedures, and use of hospital resources. Each group is assigned a cost weight based on individual-level cost accounting data from several hospitals, from which a national average can be calculated [19, 20]. The unit cost estimates for long-term residential care, home care visits, and primary care were derived from the national list for unit costs of social and health care services [21]. For all cost analyses, the national average price level and the national standard price list in 2017 were used, which were the latest versions available.

### Statistical analyses

Patient characteristics at the time of the index date are presented as numbers (n) and percentages (%) as applicable. Parameters related to opioid use are reported as medians with interquartile range (IQR) due to the high variance in the intensity of opioid use. The share of recipients who were admitted into long-term residential care or who died within the follow-up year are reported as percentages (%). We used the chi-square test to assess differences in categorical patient characteristics between home care recipients with and without dementia.

Mean annual rates for social and health care utilization per recipient are reported for the at-risk population both for the year before and the year after opioid initiation as population means. Fixed effect Poisson regressions for panel data were used to estimate the incidence rate ratios (IRR), as well as respective P-values and 95% confidence intervals (CI), for utilization rates of the seven different social and health care service categories before vs. after opioid initiation. The regression models account for differences in time at risk i.e. the decreasing size of the patient cohort, caused by mortality. P-values < 0.05 were considered statistically significant. Stata version 18.0 or later was used for all analyses.

## Results

Altogether 4,580 older adults receiving regular home care services started opioid treatment for non-cancer pain in Finland during the study period. Their mean age at the index date was 83 years (range 66 − 104) and 75.4% were female (Table 2). Musculoskeletal disorders (70.4%) and cardiovascular diseases (68.5%) were the most common chronic diseases. Of the cohort, 2.8% had recently had surgery and 6.9% had a recent injury. Within one year after the index date, 22.1% of the recipients had died and 20.8% had been permanently admitted into long-term residential care. The respective figures were 23.0% and 32.4% in opioid-initiating recipients with dementia diagnosis, and 21.7% and 16.1% in those without.

In the majority (62.8%) of cases, opioid treatment was initiated with a strong opioid, typically either transdermal buprenorphine (43.5% of all recipients) or oxycodone (16.7%). The median first opioid purchase was 252 mg OMEQ. By the end of the observation period, 71.3% of recipients with dementia had been dispensed a strong opioid, compared to 59.4% among recipients without dementia. Four-fifths of the recipients were short-term opioid users, and the remaining one-fifth extended users.

**Table 2:** Characteristics of home care recipients at the index date, and their opioid use patterns during the one-year follow-up. P values are presented for the difference between patients with vs. without dementia diagnosis

Characteristics	All *n* = 4,580	With dementia diagnosis *n* = 1,324	Without dementia diagnosis *n* = 3,256	*P*-value
	%	%	%	
Age, mean (SD)	82.8 (7.2)	83.5 (6.2)	82.5 (7.5)	
Age > 75 years	86.2	91.5	84.1	< 0.001
Gender, female	75.4	75.8	75.2	
Education				0.72
Primary	74.8	75.2	74.6	
Secondary	17.6	16.9	17.8	
University degree	7.4	7.7	7.3	
Resided in a city of > 80,000 inhabitants	20.1	20.2	20.1	0.97
> 1 home care nurse visit per day	58.4	68.5	54.4	< 0.001
Chronic diseases				
Musculoskeletal disorders	70.4	68.6	71.2	0.08
Cardiovascular diseases	68.5	64.4	70.2	< 0.001
Diabetes	24.1	19.6	25.9	< 0.001
Cerebrovascular diseases	12.2	11.6	12.5	0.43
Chronic pulmonary disease	9.8	8.1	10.5	0.01
Depression or anxiety disorder	6.6	6.9	6.4	0.49
Recent injury	6.9	7.6	6.7	0.28
Recent surgery	2.8	1.8	3.2	0.01
First opioid purchase, median OMEQ (IQR)	252 (210 − 300)	252 (225 − 375)	252 (210 − 300)	
Cumulative OMEQ/recipient/year, median (IQR)	1,008 (252 − 3,410)	1,260 (252 − 3,528)	954 (252 − 3,276)	
Started with a strong opioid	62.8	71.3	59.4	< 0.001
Duration of opioid use				
Short-term user	79.8	78.9	80.1	0.70
Extended user	20.2	21.1	19.9	0.36

*SD* standard deviation, *OMEQ *oral morphine milligram equivalents, *IQR *interquartile range

On an annual level, home care recipients had 1.76 (IRR; 95% CI 1.75 − 1.78) times more inpatient days, 1.31 (1.26 − 1.36) times more emergency care admissions, 1.09 (1.06 − 1.12) times more secondary care outpatient, and 1.02 (1.02 − 1.03) times more primary care outpatient visits after opioid initiation compared to the preceding year (Table 3). Health care service utilization of all types increased significantly in recipients without dementia, whereas the use of outpatient care services remained stable among recipients with dementia (Table 3). Home care service use decreased (0.85; 0.85 − 0.85) in recipients with dementia and increased (1.08; 1.08 − 1.08) in recipients without dementia after opioid initiation.

**Table 3:** Annual social and health care service use before and after each recipient’s opioid initiation

Social and health care service	All *n* = 4,580			
	Utilization rate before opioid initiation	Utilization rate after opioid initiation	Change in the utilization rate	*P*-value
	Mean n/recipient/year		IRR (95% CI)	
Inpatient days	20.0	31.4	1.76 (1.75–1.78)	< 0.001
Emergency care admissions	1.3	1.6	1.31 (1.26–1.36)	< 0.001
Secondary care outpatient visits	2.5	2.6	1.09 (1.06–1.12)	< 0.001
Primary care outpatient visits	27.0	27.7	1.02 (1.02–1.03)	< 0.001
Primary care phone and virtual contacts	14.2	15.4	1.11 (1.09–1.12)	< 0.001
Home care nurse visits	575.0	561.8	1.00 (1.00–1.01)	< 0.001
Days in long-term residential care	4.1	54.3	12.66 (12.47–12.86)	< 0.001

	With dementia diagnosis *n* = 1,324			
	Utilization rate before opioid initiation	Utilization rate after opioid initiation	Change in the utilization rate	P-value
	Mean n/recipient/year		IRR (95% CI)	
Inpatient days	23.3	31.7	1.49 (1.47–1.52)	< 0.001
Emergency care admissions	1.5	1.6	1.11 (1.04–1.18)	0.002
Secondary care outpatient visits	1.6	1.7	1.04 (0.98–1.11)	0.205
Primary care outpatient visits	30.4	29.6	0.96 (0.95–0.97)	< 0.001
Primary care phone and virtual contacts	15.8	15.6	0.99 (0.97–1.01)	0.425
Home care nurse visits	653.0	548.2	0.85 (0.85–0.85)	< 0.001
Days in long-term residential care	7.1	96.5	12.47 (12.20–12.74)	< 0.001

	Without dementia diagnosis *n* = 3,256			
	Utilization rate before opioid initiation	Utilization rate after opioid initiation	Change in the utilization rate	P-value
	Mean n/recipient/year		IRR (95% CI)	
Inpatient days	18.6	31.3	1.90 (1.88–1.92)	< 0.001
Emergency care admissions	1.3	1.7	1.41 (1.35–1.47)	< 0.001
Secondary care outpatient visits	2.8	3.0	1.10 (1.06–1.13)	< 0.001
Primary care outpatient visits	25.7	26.9	1.06 (1.04–1.07)	< 0.001
Primary care phone and virtual contacts	13.5	15.4	1.16 (1.14–1.17)	< 0.001
Home care nurse visits	543.3	567.3	1.08 (1.08–1.08)	< 0.001
Days in long-term residential care	2.9	37.4	12.87 (12.59–13.15)	< 0.001

**Fig. 3:**
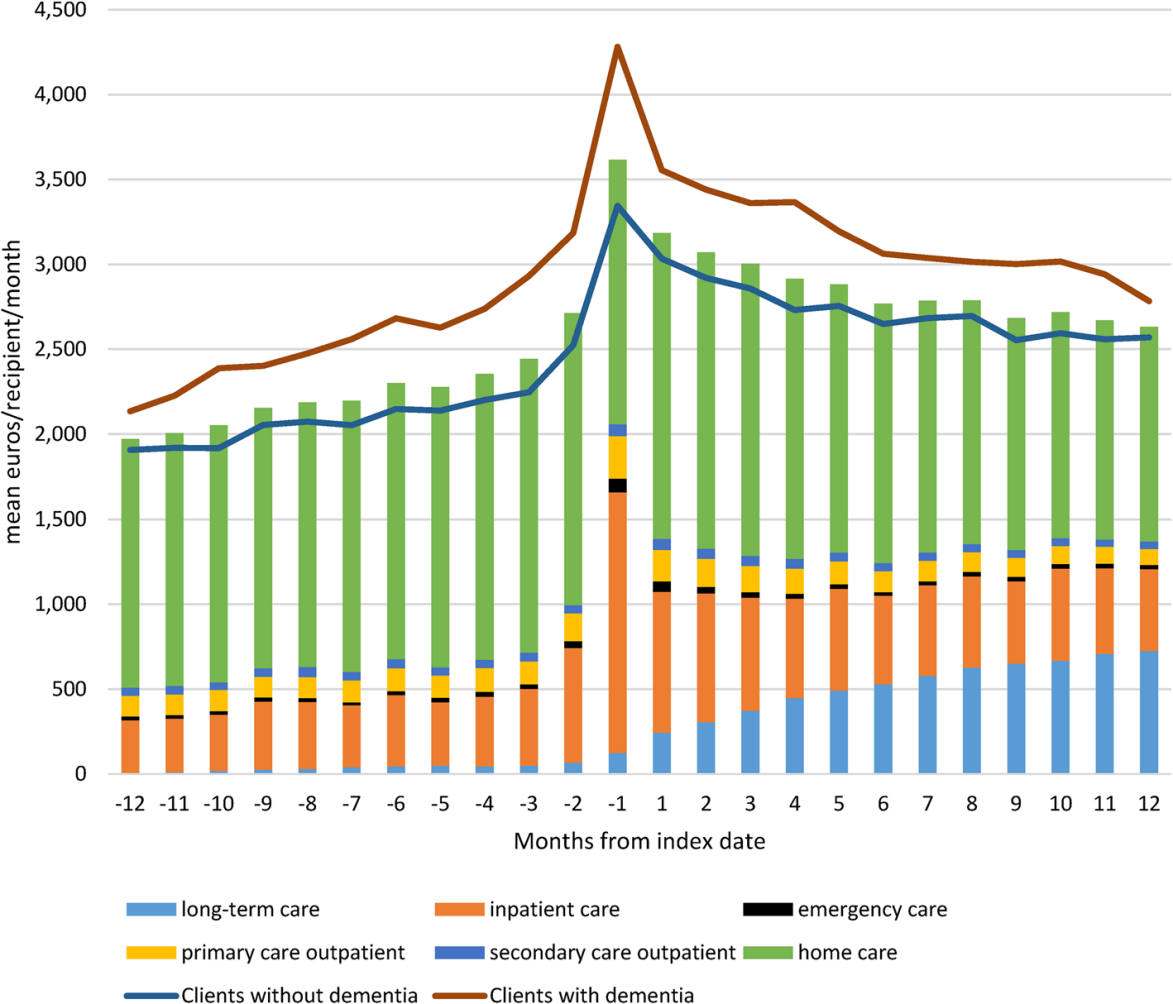
Mean monthly social and health care costs of home care recipients 365 days before and after the opioid initiation (euros/recipient/month)

The mean annual social and health care costs per recipient were 21% higher during the year following opioid initiation, compared to the costs of the preceding year (34,105 euros/recipient/year vs. 28,271 euros/recipient/year) (Fig. 3). The increase was mostly due to a shift to more heavily supported living services; at the end of the observation period, long-term residential care comprised 28% of the total monthly costs. The costs accumulated in a similar manner in recipients with and without dementia, but the absolute costs were higher in recipients with dementia.

A cost peak, consisting largely of inpatient costs, was seen during the month preceding opioid initiation, after which the total monthly costs showed a declining trend both in recipients with and without dementia, yet did not return to the original level (Fig. 3). The absolute monthly costs of inpatient and emergency care remained higher, but outpatient and home care costs were lower at the end of the year after opioid initiation compared to the first half of the pre-index year (Supplementary Table 1).

## Discussion

This study shows how older home care recipients’ social and health care use and costs changed before and after opioid initiation over a two-year observation period. Opioid initiation appeared as a marker of a change in the recipient’s health status, which was seen as a peak in health care service use, starting already before opioid initiation and lasting for a few months before stabilising. After opioid initiation, use of health and social care services was significantly greater particularly in recipients without dementia, and a significant proportion of recipients were admitted into long-term residential care during the follow-up.

The average annual number of inpatient days and emergency care admissions in home care recipients were greater after than before opioid initiation. A similar finding has been reported in persons with osteoarthritis (average age 65 years) [22] and in pre- and post-operative opioid use of more robust older adults, compared to non-users [23, 24]. Many opioid-related adverse events, such as higher rates of fall-related fractures [25], serious infections [26, 27], and mortality [28] may result in hospitalizations and emergency care admissions. These events seem to appear especially during the first few months of opioid treatment [26–28]. Consistently, inpatient and emergency care costs in the present study were higher during the first few months after opioid initiation and declined gradually. On the other hand, the association between opioid use and greater health care utilization has been stronger in persistent than short-term opioid use [4, 5]. In home care setting only long-term opioid users have been studied before, and they have had more outpatient contacts but not inpatient days compared to non-users [8]. 

Home care recipients’ average social and health care expenditures were higher after opioid initiation than during the preceding year but showed a steadily declining trend after the index date. Kern et al. [29] have previously reported a similar phenomenon of health care utilization and costs being more than two-fold immediately after opioid initiation, and then declining, yet not returning to the baseline level, in middle-aged long-term opioid users. In the present study, most recipients were short-term users.

Moreover, a rapid increase in the recipients’ expenditures could be observed already a few months before opioid initiation with a peak about a month before the index date, resulting from increased inpatient care. This indicates a significant change in some recipients’ health status before opioid initiation. Thus, probably neither adverse effects nor short-term benefits of opioids could explain the average trend of social and health care utilization even though they may affect an individual recipient’s expenditures. Instead, the changes observed are likely mostly related to acute diseases and worsened comorbidities before opioid initiation, which may have also affected physicians’ opioid prescribing. In addition, admissions to long-term residential care probably explain the gradually decreasing home care service use as well as the declining trend of health care utilization after the index date, as treatment needing hospitalizations or outpatient visits in home care can be often organized in a residential care facility with lower costs.

The average social and health care expenditures were higher in recipients with dementia than those without both before and after opioid initiation, as was expected based on previous studies [9–11]. However, the relative increase in inpatient days and emergency care admissions after opioid initiation was much higher in recipients without dementia. The recipients without dementia also had a higher number of outpatient contacts after opioid initiation whereas recipients with dementia did not. More frequent long-term residential care admissions in recipients with dementia may explain most of these differences.

The high proportion of strong opioid use in the present study was explained by the frequent use of transdermal buprenorphine. In Finland, physicians have preferred small dose transdermal buprenorphine and oxycodone in older adults, while utilization of other opioids has decreased [2].

### Strengths and limitations

The national prescription register includes all dispensed opioids in outpatient care and in practice there is no missing data. The prescription register, however, does not include drugs used in hospital setting, and thus home care recipients who have used opioids short-term during inpatient care but never purchased opioids from pharmacies are not included in the study sample. For the same reason, it was not possible to exclude inpatient opioid use during the one-year period before the index date, nor inpatient use could be included in cumulative OMEQs after opioid initiation. Misclassification of opioid use may have happened if a person decided not to use the dispensed opioid. However, home care recipients’ medications are usually managed by nurses, pharmacies, or family members, which reduces the possibility of non-adherence.

By following the same persons before and after opioid initiation, it was possible to eliminate a high number of confounding factors, which would have otherwise been difficult to detect in register-based data. The cohort included only opioid users, and therefore comparison with non-users was not possible. However, due to challenges related to confounding by indication in register-based studies, the existence of a comparison group would in any case have limited power in specifying to which extent opioid use explains the differences seen in service use and expenditures. The chosen study setting does not allow analysis of causality between opioid initiation and health and social service use. It is also possible that new diseases occurring after the index date explain the changes in health and social service use but exploring this or other associated would warrant a study of its own.

The social and health care registers used in the present study have practically complete coverage of the national population, and the data is routinely imported to the registers from electronic medical records and patient administration systems [30]. Register-based data may include inaccurate or missing coding at the level of an individual recipient, but this should not create any systematic bias. The mortality rate in the study was higher than expected and it is probable that the cohort included palliative patients despite the effort to exclude them.

## Conclusions

Hospitalizations, emergency care admissions, as well as outpatient contacts increase in home care recipients after opioid initiation, but the expenditures peak already about a month before the opioid initiation. Thus, the increased resource use may reflect mostly home care recipients’ worsened health status before opioid initiation, which likely also explains the admissions to a more heavily supported living arrangement for every fifth recipient. The present study demonstrates that comparing mean total social and health care utilization and costs before and after opioid initiation, as has been reported in previous studies, describe poorly dynamic changes in service use i.e. the shifts from one service type to another. Consequently, it can be more informative to report service use and costs by service type as a function over time instead.

Whether an older adult with dementia or frailty receives regular home care or long-term residential care can affect their total social and health care expenditure greatly, and it may also affect their use of other health care services. The relationship of opioid use and social and health care use among older adults should be further examined with methodology that accounts for potential confounding factors, such as baseline comorbidity, and conditions occurring after the initial opioid prescription.

## Supplementary Information

Supplementary Material 1.

## Data Availability

The data that support the findings of this study are available from the Finnish Social and Health Data Permit Authority Findata by request.

## Abbreviations

CI: Confidence intervals
IQR: Interquartile range
IRR: Incidence rate ratios
NSAID: Non-steroidal anti-inflammatory drugs
OMEQ: Oral morphine milligram equivalents
