# Supplementary material for: Social and health care utilization before and after opioid initiation in home care recipients with and without dementia: a nationwide register-based cohort study
Source: BMC Geriatr. 2025 Nov 3;25:833. doi: 10.1186/s12877-025-06550-z (PMC12581472; doi:10.1186/s12877-025-06550-z)
Supplement: Supplementary file 1 — Supplementary Material 1. [file 12877_2025_6550_MOESM1_ESM.docx]

Supplementary Table 1. Mean monthly costs of different services in recipients 12 months before and after opioid initiation.

|  | All (n = 4,580) | | | | | | |
| --- | --- | --- | --- | --- | --- | --- | --- |
| Months from the index date | Inpatient care | Emergency care | Primary care outpatient | Secondary care outpatient | Home care | Long-term residential care | Total costs/ month |
|  | Mean euros/ recipient | | | | | | |
| -12 | 312.2 | 21.3 | 122.0 | 49.4 | 1461.1 | 5.2 | 1971.3 |
| -11 | 315.2 | 21.3 | 122.9 | 50.1 | 1486.3 | 10.2 | 2006.0 |
| -10 | 330.0 | 22.4 | 123.3 | 45.6 | 1511.0 | 19.1 | 2051.4 |
| -9 | 399.1 | 25.8 | 121.4 | 49.7 | 1530.3 | 26.8 | 2153.1 |
| -8 | 393.1 | 23.6 | 124.6 | 58.1 | 1557.8 | 30.5 | 2187.6 |
| -7 | 365.5 | 17.8 | 128.8 | 51.8 | 1594.4 | 38.9 | 2197.1 |
| -6 | 418.9 | 24.2 | 134.5 | 54.3 | 1622.5 | 45.8 | 2300.2 |
| -5 | 374.5 | 26.5 | 131.3 | 48.5 | 1649.1 | 48.1 | 2277.9 |
| -4 | 408.9 | 29.0 | 140.7 | 48.0 | 1682.0 | 46.1 | 2354.7 |
| -3 | 452.3 | 26.0 | 135.7 | 51.1 | 1727.6 | 49.8 | 2442.5 |
| -2 | 676.5 | 39.4 | 163.8 | 49.1 | 1717.9 | 66.6 | 2713.2 |
| -1 | 1532.5 | 82.0 | 249.4 | 68.8 | 1558.9 | 124.1 | 3615.8 |
| 1 | 830.0 | 61.5 | 184.2 | 66.7 | 1797.8 | 244.2 | 3184.4 |
| 2 | 759.1 | 39.0 | 165.9 | 58.7 | 1744.4 | 304.3 | 3071.4 |
| 3 | 666.4 | 33.0 | 155.3 | 58.5 | 1719.1 | 372.0 | 3004.4 |
| 4 | 586.3 | 28.4 | 149.5 | 56.3 | 1648.4 | 447.1 | 2915.9 |
| 5 | 599.3 | 27.0 | 133.8 | 52.7 | 1577.9 | 492.0 | 2882.6 |
| 6 | 521.9 | 22.0 | 123.4 | 47.5 | 1525.3 | 528.5 | 2768.5 |
| 7 | 534.5 | 22.9 | 120.2 | 49.3 | 1480.7 | 578.4 | 2786.1 |
| 8 | 536.5 | 28.1 | 115.9 | 48.4 | 1432.6 | 627.1 | 2788.5 |
| 9 | 486.4 | 27.0 | 111.5 | 46.3 | 1363.2 | 649.6 | 2684.0 |
| 10 | 543.7 | 25.2 | 106.2 | 46.8 | 1328.3 | 667.5 | 2717.6 |
| 11 | 504.6 | 25.2 | 101.5 | 41.4 | 1288.1 | 708.7 | 2669.6 |
| 12 | 482.0 | 25.4 | 93.5 | 44.9 | 1260.5 | 725.4 | 2631.5 |
